# Naringin Release from a Nano-Hydroxyapatite/Collagen Scaffold Promotes Osteogenesis and Bone Tissue Reconstruction

**DOI:** 10.3390/polym14163260

**Published:** 2022-08-10

**Authors:** Yanping Zuo, Qiwen Li, Qiuchan Xiong, Jing Li, Chengfang Tang, Yaochao Zhang, Danyang Wang

**Affiliations:** 1 Department of Prosthodontics, School of Stomatology, Xi’an Medical University, Xi’an 710021, China; 2 State Key Laboratory of Oral Diseases, National Clinical Research Center for Oral Diseases, West China Hospital of Stomatology, Sichuan University, Chengdu 610041, China; 3 Department of Cardiothoracic Surgery, University Medical Center Regensburg, 93053 Regensburg, Germany

**Keywords:** bone tissue reconstruction, naringin, hydroxyapatite, scaffold, collagen, osteogenesis

## Abstract

Bone fractures and defects are a major health issue and have reportedly affected over 455 million individuals globally to date. Bone tissue engineering has gained great success in bone defect repair and bone reconstruction based on the use of nano-hydroxyapatite (nHA) or collagen (COL). Both nHA and COL exhibit osteogenic induction capacity to support bone tissue regeneration; however, the former suffers from poor flexibility and the latter lacks mechanical strength. Biological scaffolds created by combining nHA and COL (nHA/COL) can overcome the drawbacks imposed by individual materials and, therefore, have become widely applied in tissue engineering. The composite scaffolds can further promote tissue reconstruction by allowing the loading of various growth factors. Naringin (NG) is a natural flavonoid. Its molecular weight is 580.53 Da, lower than that of many growth factors, and it causes minimal immune responses when being introduced in vivo. In addition, naringin is safe, non-toxic, inexpensive to produce, and has superior bio-properties. In this study, we introduced NG into a nHA/COL scaffold (NG/nHA/COL) and exploited the potentials of the NG/nHA/COL scaffold in enhancing bone tissue regeneration. NG/nHA/COL scaffolds were fabricated by firstly combining nHA and collagen at different compositional ratios, followed by NG encapsulation. NG release tests showed that the scaffold with a nHA/COL mass ratio of 7:3 exhibited the optimal property. The in vitro cell study showed the desirable biocompatibility of the NG/nHA/COL scaffold, and its effective promotion for the osteogenic differentiation of bone marrow mesenchymal stem cells (BMSCs), as proved by an increased alkaline phosphatase (ALP) activity, the formation of more calcium nodules, and a higher expression of osteogenic-related genes involving *Osteocalcin* (*OCN*), *BMP-2*, and *Osteopontin* (*OPN*), compared with the control and nHA/COL groups. When administered into rats with skull defects, the NG/nHA/COL scaffold significantly promoted the reconstruction of bone tissues and the early repair of skull defects, indicating the great potential of NG/nHA/COL scaffolds in bone tissue engineering.

## 1. Introduction

Traditional therapeutic methods such as bone autograft and allograft for the repair of bone defects have limited clinical applications due to the low amount of autologous bone, additional trauma, infection, pain, allogeneic tissue rejection and other complications in patients [1,2,3]. Alternatively, the use of bioactive materials has shown great potentials in bone tissue repair and bone reconstruction. Three-dimensional (3D) scaffold biomaterials containing bioactive components such as cells and growth factors are desirable in bone tissue engineering, as they can release active components locally, construct a biomimetic structural environment, induce the formation of an extracellular matrix (ECM), promote the adhesion and growth of cells, and stimulate the physiological repair process.

Nano-hydroxyapatite (nHA) is the main component of hard tissue and is characterized by good biocompatibility, osteo-conductivity, and bioactivity. Several previous studies on nHA have confirmed its large specific surface, good stability, biocompatibility, and excellent bone conductivity, thereby making it a good scaffold material in tissue engineering [4,5,6]. However, its low absorption rate may impede its degradation in vivo and delay the process of new bone formation [7]. Furthermore, its inherent hardness, brittleness, and lack of flexibility also limit its clinical application [8]. Collagen (COL), as a natural polymer material derived from organisms, has good biocompatibility and degradation characteristics [9,10]. Collagen scaffolds can be used as carriers for cells and growth factors without affecting their bioactivity to promote tissue reconstruction [11]. The fibrous structure formed by collagen-based hydrogels shows good biochemical properties and rapid activation of cellular responses due to its natural ECM-like structure. However, COL has poor mechanical strength and is susceptible to fast absorption. Therefore, the combination of nHA and COL as scaffold materials can fully utilize their advantages for bone conduction and bone induction and optimize the physical and chemical properties of scaffold materials. Previous studies have reported that COL and hydroxyapatite can induce osteogenesis differentiation of bone marrow mesenchymal stem cells (BMSCs) [12]. Mingming Ou et al. [13] reported the preparation of nHA/COL scaffolds with different nHA and COL composition ratios and that nHA/COL with a mass ratio of 7:3 showed the best osteogenesis effects among all others.

Bioactive molecules are key elements in scaffold materials as they can enhance the function of biomaterials and promote the aggregation and differentiation of osteoblasts in bone tissue engineering. However, applications of many currently studied osteogenic active molecules have been limited because of their high production costs and potential side effects. In contrast, some natural substances have gained increasing attention due to their high availability, low production costs, and excellent biological activity. Naringin (NG) is a dihydroflavonoid and is mainly found in the skins and flesh of *Citrus grandis* (L.) Osbeck. It is also the main active ingredient of the Chinese medicine rhizome. The osteogenic induction, anti-absorbance, and anti-adipogenesis properties of NG make it a desirable biomaterial in bone tissue engineering [14]. It has been shown in several studies that NG can promote proliferation and osteogenesis differentiation of BMSCs [15,16,17,18]. Further studies have found that NG can promote the osteogenic differentiation of human BMSCs by the upregulation of microRNA-20a, down-regulation of PPARγ [19], and activation of the ERK signal [20], as well as by the upregulation of *Foxc2* expression through the IHH signaling pathway [21] and activation of the Notch signaling pathway [22]. Wong et al. [23] established a rabbit skull defect model and demonstrated the effects of NG, in combination with COL, on promoting the formation of new bone. A variety of naringin-containing biological materials, including electrospun PLGA/PLLA/PDLLA blend fibers loaded with NG [24], an electrosprayed naringin-loaded microsphere/SAIB hybrid [25], a naringin-inlaid silk fibroin/hydroxyapatite scaffold [26], and a naringin-contained porous gelatin composite [17] have been studied to explore the osteogenic properties of naringin in a closely mimicked ECM. NG has shown great promise for use as a scaffold material for promoting stem cell differentiation toward pro-osteogenic phenotype and other bone diseases [14].

**Figure 1:**
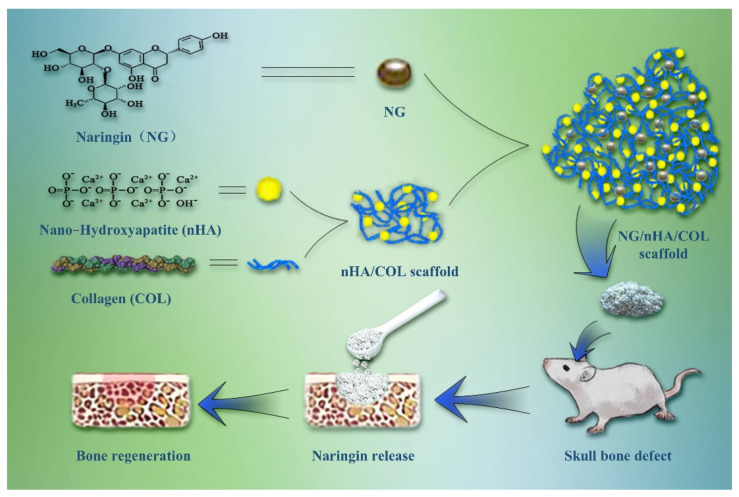
Schematic illustration of NG/nHA/COL scaffold construction and the pipeline of NG/nHA/COL scaffold applied for bone regeneration.

In the present study, we designed and fabricated a NG/nHA/COL composite scaffold and investigated its efficacy for promoting osteogenesis and bone tissue reconstruction both in vitro and in vivo. The loading and encapsulation efficiency of NG and its cumulative release rate were evaluated to determine the optimal nHA/COL ratios. Compositional and structural properties and biocompatibility of the scaffold materials were characterized. The NG/nHA /COL scaffold was then embedded into a bone defect area in a rat model, as illustrated in Figure 1. Three-dimensional (3D) reconstruction data, micro computed tomography (µCT), and histological section staining of the skull bone defect were used to evaluate the osteogenic properties of the scaffold materials.

The ideal scaffold material for bone tissue engineering should have good mechanical strength and provide support for the new tissue. R.W.K. Wong et al. [23] demonstrated the positive role of naringin collagen in the repair of 5 mm × 10 mm bone defects in vivo. In the present study, we also verified the effectiveness of NG/nHA/COL in repairing critical bone defects. These results indicate that the composites with naringin have good mechanical properties.

## 2. Materials and Methods

### 2.1. Fabrication of Scaffolds

To fabricate the nHA/COL scaffold, 100 mg collagen type I was dissolved in 2% acetic acid solution (pH = 4) in a 4 °C water bath. The pH was then adjusted to 7.2 ± 0.2 after full stirring to generate a neutral collagen solution. Amounts of 1.8 g, 1.6 g, 1.4 g, and 1.2 g nHA powder were weighed and evenly added to the 10 mL collagen solution in the 4 °C water bath. After 30 min of ultrasonication, samples were stored at 25 °C for 16–20 h, followed by centrifugation and being washed more than thrice. Vacuum freeze-drying was then performed to obtain nHA/COL scaffolds, resulting in nHA/COL mass ratios of 9:1, 8:2, 7:3, and 6:4.

To prepare NG/nHA/COL scaffolds, 1.5 g NG was fully dissolved in 300 g ethanol to obtain naringin solution (mass ratio 0.5%), which was then mixed with nHA/COL obtained at different mass ratios. The solution was ultrasonicated for 15 min and stirred for 3 h. Ethanol was volatilized at 45 °C, and the solution was ventilated and dried in an ultra-clean platform for 8 h, followed by UV irradiation for 3 h and vacuum-drying for 3 h to produce NG/nHA/COL.

### 2.2. Characterization of Scaffolds

#### 2.2.1. Loading Rate and Encapsulation Rate of Scaffolds

Spectrophotometric values of NG with different concentrations (500, 100, 50, 20, 10, 1 μg/L) were determined using a UV spectrophotometer at 287 nm, and the standard curve was made. The drug loading rate of the scaffold material entails the percentage of drug content in the total material mass. Ten milligrams of NG/nHA/COL dry mass was accurately weighed and fully dissolved in 4 mL dichloromethane. The resulting material was dissolved under ultrasound and NG was fully released by an ultrasonic cell pulverizer. NG in dichloromethane was extracted with 10 mL methanol solution, transferred to rapid mixer for full shock, and incubated for 10 min. The resulting supernatant was centrifuged at room temperature as a sample solution. The absorbance was then determined at 287 nm. The total mass of NG contained in the scaffold material was computed using the NG standard curve equation. Drug loading and encapsulation rates were calculated using the following formulae:

$$\mathrm{Drug}\;\mathrm{loading}\;\mathrm{rate}=\left(\frac{\mathrm{total}\;\mathrm{drug}\;\mathrm{mass}\;\mathrm{in}\;\mathrm{stents}}{\mathrm{total}\;\mathrm{stent}\;\mathrm{mass}}\right)\times 100\%$$

$$\mathrm{Encapsulation}\;\mathrm{rate}=\left(\frac{\mathrm{total}\;\mathrm{drug}\;\mathrm{mass}\;\mathrm{in}\;\mathrm{stents}}{\mathrm{total}\;\mathrm{drug}\;\mathrm{mass}\;\mathrm{input}}\right)\times 100\%$$

#### 2.2.2. Release Profile of Scaffolds In Vitro

Ten milligrams of NG/nHA/COL with varying nHA/COL mass ratios was immersed in 10 mL PBS and stirred in an oscillator maintained at 37 °C. Supernatant was then collected at different time intervals (1, 2, 4, 6, 8, 10, 12, 24, 36, and 48 h, and 3, 5, 6 7, 8, 12, and 14 d), and the absorbance of the resulting solution was determined using the UV spectrophotometer at 287 nm after centrifugation at 8000 rpm. The cumulative release rate was calculated based on the standard curve.

The optimal mass ratio of NG/nHA/COL was obtained based on the loading, encapsulation, and cumulative release rates of NG and was selected for preparing scaffolds for the subsequent in vitro and in vivo experiments.

#### 2.2.3. SEM Image of Scaffolds

The conductive adhesive was bonded to the sample holder, and an appropriate amount of materials of each group was ground into powder, which was evenly sprinkled on the conductive adhesive. Then, the unglued sample was gently blown off with an ear washing ball, and the surface structure and morphology of the scaffold materials were inspected using a scanning electron microscope (SEM).

#### 2.2.4. FTIR of Scaffolds

A Fourier infrared (FTIR) spectrometer was used to determine sample composition (wave number range of 4000–400 cm^−1^, scanning of 32 times, and resolution of 4 cm^−1^). The materials of each group were fully dried and ground into fine powder. A small amount of sample (1–2 mg) was mixed with 200 mg of pure KBr and ground evenly, then placed in the mold and pressed into thin sections with a tablet press. Then, the sample was put into the FTIR spectrometer for testing.

### 2.3. Bioactivity In Vitro

#### 2.3.1. Cell Viability and Proliferation Assays

The nHA/COL mass ratio of 7:3 was selected for preparing nHA/COL for subsequent cell and animal experiments. In cell studies, 0.1 g of Co60-sterilized nHA/COL and NG/nHA/COL powders were placed in 10 mL culture medium or osteogenic induction medium at room temperature for 24 h and centrifugated at 1000 rpm to obtain supernatant. Then, a 0.22 μm filter was used to filter and sterilize the supernatant for cell experiments. The osteogenic induction culture medium used in this study was composed of dexamethasone (Solarbio, Beijing, China), ascorbic acid (Solarbio, Beijing, China), and β-glycerophosphate (Sigma, St. Louis, MO, USA).

Primary BMSCs were extracted from 3- to 4-week-old SD rats. The cells were plated in culture flasks and incubated in 5% CO_2_ and 95% relative humidity at 37 °C, and then were cultured in Dulbecco’s modified Eagle’s medium (DMEM, HyClone, Logan, UT, USA) supplemented with 100 U/mL penicillin/streptomycin (HyClone, Logan, UT, USA) and 10% fetal bovine serum (FBS, Gibco, Waltham, MA, USA). Third-generation BMSCs were then harvested for subsequent experiments.

Cell proliferation and morphology were assessed as follows: We seeded BMSCs in 96-well culture plates with 3 × 10^3^ cells per well, with each well containing 100 μL of culture medium. The cells were then cultured for 24 h and replaced with serum-free DMEM medium for 24 h. After cell synchronization, control group medium was replaced with culture medium, and experimental groups were replaced with nHA/COL and NG/nHA/COL culture media extracts, while drug-containing blank culture medium (without cells) served as zero adjustment wells. Following cell incubation for 24 h, Cytotoxicity (Beyotime, Shanghai, China) and Calcein/PI Cell Activity Assay Kits were used for live/dead cell staining. The cytoskeleton and nucleus were stained with phalloidin and DAPI (Beyotime, Shanghai, China), and the distribution of live and dead cells as well as cell morphology were observed using fluorescence microscopy (IX73, OLYMPUS). After 1, 3, 5, and 7 d of cell culture, cell proliferation was determined by Cell Counting Kit-8 (CCK-8) (APExBIO Technology LLC, Houston, TX, USA) Assay. Medium containing 10%CCK-8 staining solution was prepared first, then 100 μL of the prepared medium was added to each well. The absorbance of each well was measured at 450 nm using a microplate spectrometer (Thermo Scientific, Waltham, MA, USA) after incubation for 3 h in the incubator. This experiment was repeated thrice and mean values were computed.

#### 2.3.2. In Vitro Osteogenic Differentiation

BMSCs suspension with a density of 3 × 10^4^ in 24-well plates cultured for 24 h were used for the evaluation of osteogenic differentiation potential. After cell synchronization, the control group was replaced with osteogenic induction solution and the experimental group was replaced with nHA/COL and NG/nHA/COL osteogenic induction extracts. Media were replaced every 3 days (n = 3).

Alkaline phosphatase is an exoenzyme of osteoblast, and its expression activity is an obvious characteristic of osteoblast differentiation. The cells were firstly induced into osteogenesis for 7 days, and then the protein was extracted and its concentration was tested using a BCA kit (Beyotime, Shanghai, China); then, the ALP activity was tested using an ALP activity detection kit (Beyotime, Shanghai, China). At the same time, ALP staining was performed using an Alkaline Phosphatase Staining kit (Beyotime, Shanghai, China). A microscope (IX73, OLYMPUS) was used to take staining microscopic images.

After 21 d of osteogenic induction, alizarin Red S (Cyagen Biosciences (Suzhou) Inc., China) was used to stain cells for 5 min. After images were taken with a microscope, 5% perchloric acid was used to extract the ARS dye and a microplate spectrometer (Thermo Scientific, Waltham, MA, USA) was used to determine the absorbance at 450 nm.

Six-well plates were used to detect the expression of osteogenic-related genes; 2.0 mL of BMSCs suspension with a density of 3 × 10^4^ was cultured per well. After cell synchronization, the control group was replaced with osteogenic induction solution, experimental groups were replaced with nHA/COL and NG/nHA/COL osteogenic induction extracts, and the media was replaced every 3 days (n = 3). Cellular RNA was extracted and reversed after 7 days of osteogenic induction. The expression of osteogenic-related genes Runx2, BMP-2, OCN, and OPN was detected by a real-time quantitative qPCR instrument (Roche, Basel, Switzerland) using the 2^−ΔΔ CT^ method. Primers for target genes Runx2, BMP-2, OCN, and OPN and internal reference gene GAPDH were designed based on sequences published by GenBank.

### 2.4. In Vivo Animal Experiment

Osteoblasts are obtained through the proliferation, growth, and maturation of osteoblast precursor cells. Several animal models have been used to evaluate the effect of bone regeneration, including skull defects, long or segmental bone defects, partial cortical defects, and cancellous bone defects [27,28]. The skull defect model is a commonly used model to evaluate the osteogenic performance of bioactive material [29].

The current study used male SD rats (310 g ± 28 g) as animal models of bone defect. We performed the animal experiments in accordance with the Guide for Care and Use of Laboratory Animals from the National Institutes of Health, and the protocol was approved by the Ethics Committee of Xi’an Medical University (XYLS2021219). Simply stated, a cylindrical defect of 2 mm diameter and 2 mm height was created using Kirschner wire on either side of the medial cranial suture of the skull. The sites of bone defect were randomly implanted with sterilized nHA/COL and NG/nHA/COL scaffolds. After the surgery, penicillin was injected intramuscularly for three days to prevent infection.

All SD rats were sacrificed 4 weeks after surgery, and the calvarias samples were collected. Bone samples were fixed with 10% paraformaldehyde and stored in PBS at 4 °C. They were scanned at 10 μm intervals using μCT50 (SCANCO Medical AG, Basseldorf, Switzerland). Samples were reconstructed in three dimensions (3D), and the trabecular number (Tb.N), bone volume/total volume (BV/TV), and mean/density of the TV in the defect area were calculated. After that, samples were fixed at room temperature for 4 weeks using ethylene diamine tetraacetic acid (EDTA) (VWR, Radnor, PA, USA) solution. Dehydration, embedding, and slicing were performed to obtain the hard tissue sections. Masson’s trichrome (Solarbio, Beijing, China) and hematoxylin–eosin (H&E) (Solarbio, Beijing, China) staining were used to observe new tissue in the bone defect area. Images were taken under a light microscope (DP73, OLYMPUS).

## 3. Results and Discussion

### 3.1. Preparation and Characterization of Scaffolds

**Figure 2:**
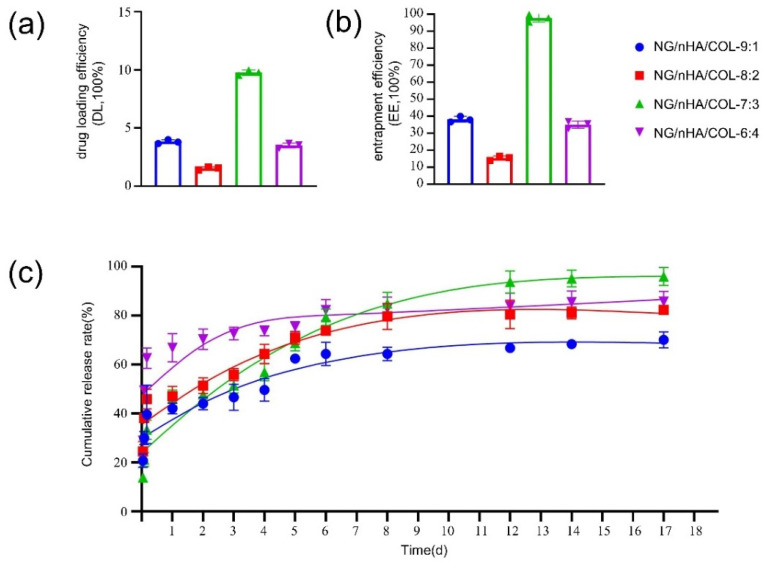
Drug loading efficiency, entrapment efficiency, and cumulative release rate of NG/nHA/COL: (**a**) drug loading efficiency (DL, 100%) of NG/nHA/COL, (**b**) entrapment efficiency (EE, 100%) of NG/nHA/COL, and (**c**) cumulative release rate (%) of naringin released from NG/nHA/COL.

Our findings showed that the best drug loading (Figure 2a) and encapsulation rates (Figure 2b) of NG can be obtained at the nHAP/COL mass ratio of 7:3. The results of the accumulative release rate (Figure 2c) showed that NG release increased rapidly from day 1 to day 2. A peak of the release rate was first observed in the mass ratio 6:4 group until it reached a platform after day 4. NG release in the 7:3 group was lower than that of the others within the first 2 days, but slowly increased in a steady and sustainable way to take a leading position after day 7 and throughout our long-term observation period up to 17 days. Based on the findings of drug loading, encapsulation, and accumulative release rates, nHA/COL with a 7:3 mass ratio was selected for subsequent experiments.

**Figure 3:**
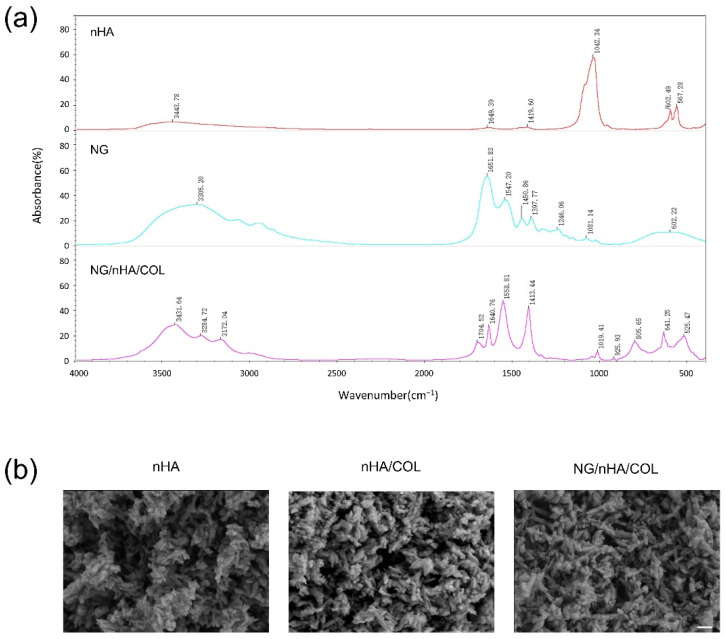
FTIR and SEM of scaffolds: (**a**) FTIR of nHA, NG, and NG/nHA/COL and (**b**) SEM of nHA, nHA/COL, and NG/nHA/COL; the mass ratio of nHA/COL is 7:3 (scale bar: 200 nm).

The FTIR spectrum of nHA indicated a characteristic absorption peak at 3443 cm^−1^ by O–H stretching vibration. The characteristic absorption peak at 1649 cm^−1^ is assigned to H_2_O. Characteristic absorption peaks at 602 cm^−1^ and 567 cm^−1^ are assigned to PO_4_^3−^. The FTIR spectrum of NG/nHA/COL showed that characteristic absorption peaks at 1559 cm^−1^ and 1413 cm^−1^ were derived from the benzene ring in naringin. Moreover, characteristic absorption peaks at 1019 cm^−1^, 925 cm^−1^, 641 cm^−1^, and 524 cm^−1^ were derived from PO_4_^3−^ in nHA and benzene rings. In addition, the peak at 1641 cm^−1^ was formed by the superposition of the NH_2_ amide peak I (ν_C =O_) of collagen and C=O of the C ring of NG, and the characteristic absorption peak of 3450~3100 cm^−1^ was formed by the superposition of the stretching vibration of –OH in nHA and NG. These findings demonstrated the presence of characteristic absorption peaks of NG, nHA, and COL in the spectrum of the NG/nHA/COL scaffold, and no new covalent bonds were identified, indicating that the basic chemical structures of naringin and nHA/COL were well preserved after synthesis (Figure 3a).

SEM images revealed the porous spongy structure of nHA/COL scaffolds with different ratios. The porous spongy structure of the scaffolds did not change after introducing naringin into them (Figure 3b).

### 3.2. The NG/nHA/COL Scaffold Promoted the Proliferation of BMSCs

**Figure 4:**
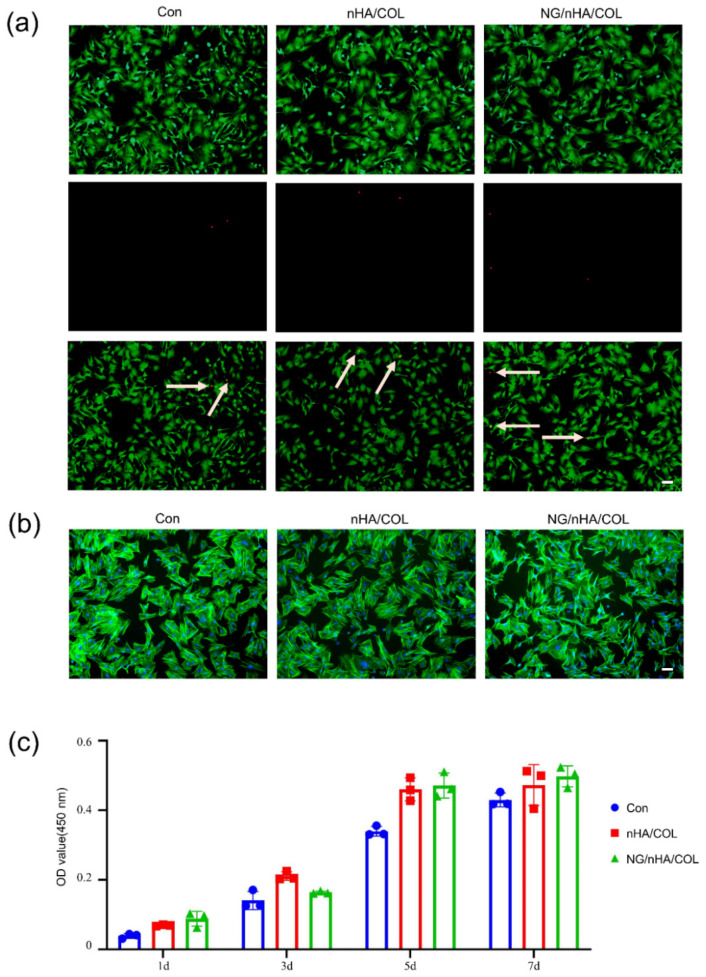
In vitro biocompatibility investigation of NG/nHA/COL. BMSCs were cultured with nHA/COL and NG/nHA/COL culture media extracts. (**a**) After 24 h incubation, the live/dead staining of BMSCs in control, nHA/COL, and NG/nHA/COL groups (scale bar: 200 µm). (**b**) After 24 h incubation, phalloidin and DAPI proliferation staining of BMSCs in control, nHA/COL, and NG/nHA/COL groups (scale bar: 200 µm). (**c**) Proliferation of BMSCs of control, nHA/COL, and NG/nHA/COL groups analyzed by CCK-8 kit (n = 3).

Live and dead staining after 24 h culture showed a minimal number of dead cells (red) in all three study groups (control, nHA/COL, and NG/nHA/COL), and that live cells (green) were evenly distributed across the entire field of view (Figure 4a). Cell scaffold/nucleus staining revealed spindle-shaped cells in all three groups, where the cell nuclei were brightly stained, and no significant differences in cell morphology were noticed (Figure 4b). The CCK-8 kit was used to quantitatively evaluate the cytotoxicity of the scaffold materials. Our findings showed that cells of all three groups underwent a certain degree of proliferation by being given an extended culture time, and more pronounced cell proliferation was observed in both the nHA/COL and NG/nHA/COL groups compared with the control group (Figure 4c).

### 3.3. The NG/nHA/COL Scaffold Promoted the Osteogenic Differentiation of BMSCs

**Figure 5:**
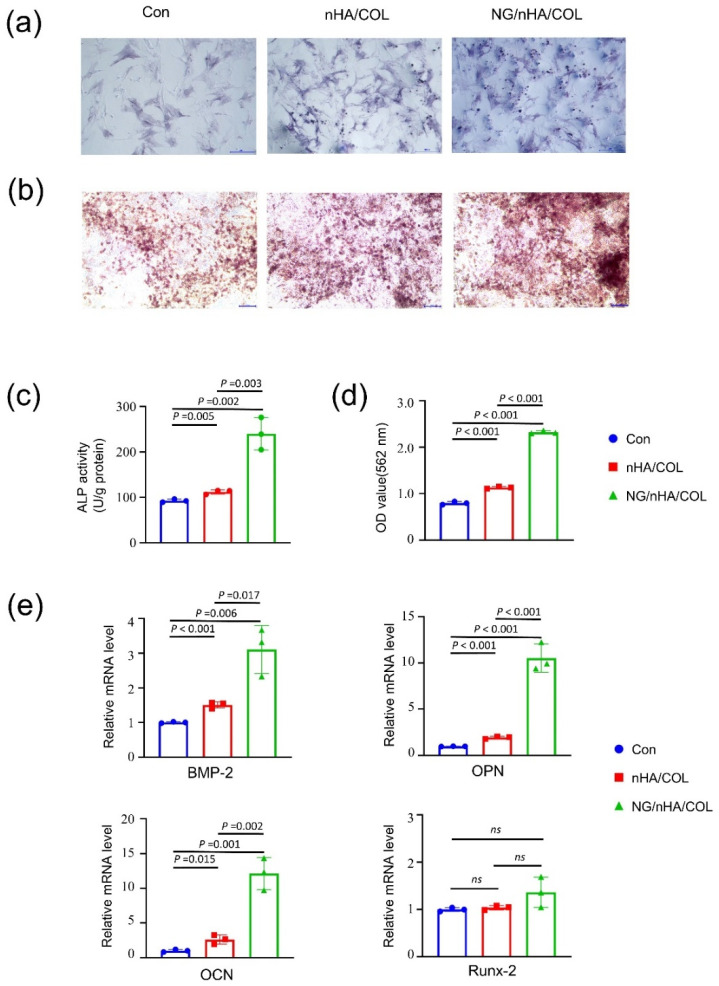
In vitro osteogenesis properties of NG/nHA/COL. BMSCs were cultured using nHA/COL and NG/nHA/COL culture media extracts. (**a**) ALP staining images of control, nHA/COL, and NG/nHA/COL groups after 7 d of osteogenic induction (scale bar: 200 µm). (**b**) ARS staining images of control, nHA/COL, and NG/nHA/COL groups after 21 d of osteogenic induction (scale bar: 100 µm). (**c**) ALP activity of three groups (n = 3). (**d**) Quantitative results of ARS staining of three groups (n = 3). (**e**) The osteogenic-related genes expression, including BMP-2, OPN, OCN, and Runx-2 (n = 3).

After 7 days of osteogenesis induction, ALP staining on BMSCs indicated that the cells in the NG/nHA/COL group had more prominent ALP activity than that in the control and nHA/COL groups (Figure 5a). The ALP activity in the NG/nHA/COL group (240.24 ± 29.11 U g^−1^ protein) was higher than that of the control (92.84 ± 2.82 U g^−1^ protein) and nHA/COL (112.09 ± 3.81 U g^−1^ protein) groups as shown by the quantitative analyses (Figure 5c). ARS staining was used as a marker of calcium deposition to analyze the mineralization efficiency, and the enhanced formation of calcified nodules was observed in the NG/nHA/COL group 21 days after osteogenic induction (Figure 5b). Quantification showed a higher level of ARS in the NG/nHA/COL group than that in the nHA/COL and control groups (Figure 5d) (*p* < 0.05). These findings demonstrate the effectiveness of NG/nHA/COL in promoting the osteogenic differentiation of BMSCs.

We then quantified the efficacy of scaffolds for enhancing osteogenic differentiation by detecting the expression of osteoblast-related genes after osteogenic induction for 7 days. Our findings showed that the expression levels of OCN, BMP-2, and OPN in the NG/nHA/COL group were upregulated 12.14 ± 1.89-fold (*p* < 0.05), 3.11 ± 0.57-fold (*p* < 0.05), and 10.54 ± 1.24-fold (*p* < 0.05), respectively, and these expressions were also significantly higher than those in the nHA/COL group (*p* < 0.05) (Figure 5e). These results indicate that nHA/COL, in combination with naringin, dramatically increased the expression of osteogenic genes in BMSCs. However, the upregulation of the expression level of Runx2 was insignificant in the experimental groups, which is consistent with the results of Qiao Y. et al. [30], indicating the role of Runx2 in earlier osteoblastic differentiation. The qPCR results showed that naringin released from the NG/nHA/COL scaffold significantly promoted the osteogenic differentiation of BMSCs by enhancing the expression of osteoblast-related genes.

### 3.4. The NG/nHA/COL Scaffold Promoted the Repair of Rat Skull Defect

**Figure 6:**
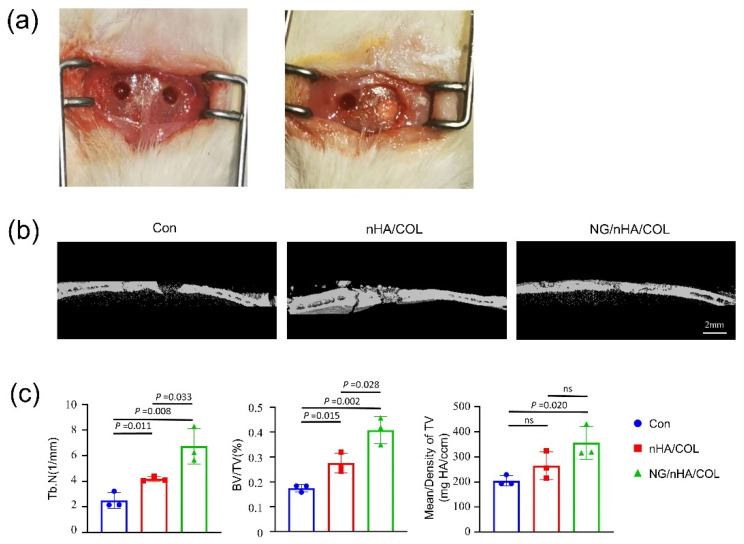
In vivo osteogenesis properties of NG/nHA/COL. The nHA/COL and NG/nHA/COL scaffolds were implanted to the skull defect areas of SD rats. (**a**) Skull defect model in SD rats. (**b**) Three-dimensional reconstruction image of skull defect areas using µCT scanning (scale bar: 2 mm). (**c**) BV/TV, Tb.N, and mean/density of skull defect areas (n = 3).

We verified the effects of NG/nHA/COL on the early osteogenesis (4 W) of skull defect in SD rats by establishing a critical bone skull defect rat model (Figure 6a). Three-dimensional reconstruction and evaluation of cranial parietal defect was performed using μCT after 4 weeks of imbedding the control and experimental groups. A higher mass of new bone was found in the NG/nHA/COL and nHA/COL groups compared with that of the control group. More importantly, the density of new bone in the NG/nHA/COL group was higher than that in the nHA/COL group (Figure 6b).

The quantitative analysis of the new bone showed that the BV/TV of the NG/nHA/COL group was 40.80%, which was higher than that of the control and nHA/COL groups (NG/nHA/COL vs. nHA/COL, *p* < 0.05; NG/nHA/COL vs. control, *p* < 0.05; nHA/COL vs. control, *p* < 0.05) (Figure 6c). The trabecular number (Tb.N) in the NG/nHA/COL group was 6.73 ± 1.12/ mm, which was also higher than that of the control and nHA/COL groups (NG/nHA/COL vs. nHA/COL, *p* < 0.05; NG/nHA/COL vs. control, *p* < 0.05; nHA/COL vs. control, *p* < 0.05). These findings indicate that the NG/nHA/COL scaffold can accelerate the bone healing process (Figure 6c). 

**Figure 7:**
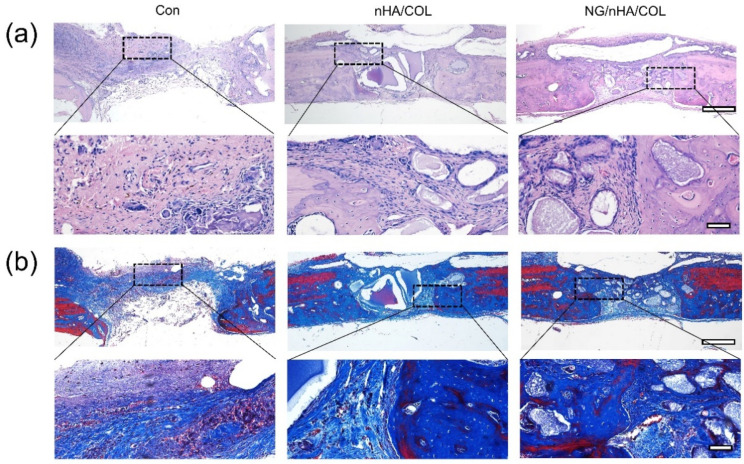
Histomorphological observation of bone defect areas by staining hard tissue section: (**a**) H&E staining and (**b**) Masson’s trichrome staining (scale bar: 200 µm and 40 µm).

In good agreement with μCT, the results of Masson’s and H&E trichrome staining indicated different degrees of the bone repair of defects in the three groups. In the control group, a large amount of fibrous connective tissue was observed in defect areas, at the edges of which was a small amount of new bone tissue. In the NG/nHA/COL group, many new bone trabeculae were formed at the defect sites with the presence of osteoblasts. Although new bone formation was found in both experimental groups, the mass of new bone was significantly higher in the NG/nHA/COL than in the nHA/COL group. These findings further demonstrate that the reconstruction and regeneration of bone tissue in defect areas can be promoted effectively by the NG/nHA/COL scaffold, thanks to the biological and pharmacological properties of NG and its continuous and sustained release from the NG/nHA/COL composite scaffold (Figure 7).

## 4. Conclusions

In conclusion, we fabricated a NG/nHA/COL scaffold by loading naringin, a natural glycoside with excellent biological and pharmacological properties, to a nHA/COL scaffold for promoting the healing process of bone defect, and conducted characterizations with various analytical methods as well as cell and animal studies. NG loading, encapsulating, and cumulative release rates were assessed to obtain the optimal nHA/COL mass ratio of 7:3. FTIR and SEM revealed the compositional and structural integrity of the scaffolds before and after synthesis. Cytotoxicity and proliferation studies using BMSCs confirmed the good biocompatibility of NG/nHA/COL. In addition, cell osteogenesis experiments showed that NG/nHA/COL significantly enhanced ALP phosphatase activity, which effectively induced the formation of calcium nodules and increased the expression of osteogenic-related genes in BMSCs. In vivo, NG/nHA/COL led to a significant regeneration of bone tissues (BV/TV = 40.80%) in the rat skull defect area within 4 weeks, indicating the superior pharmacological properties of naringin and its sustainable release from the NG/nHA/COL scaffold for accelerating the healing. Our findings suggest the great potential of using NG/nHA/COL scaffolds as an alternative to bone transplantation for the repair of bone defects in future laboratory and clinical applications.
